# Supplementary material for: Development of a Low-Molecular-Weight Aβ42 Detection System Using a Enzyme-Linked Peptide Assay
Source: Biomolecules. 2021 Dec 2;11(12):1818. doi: 10.3390/biom11121818 (PMC8699310; doi:10.3390/biom11121818)
Supplement: Supplementary file 1 [file biomolecules-11-01818-s001.zip › biomolecules-1456561-supplementary.pdf]

Supplementary data

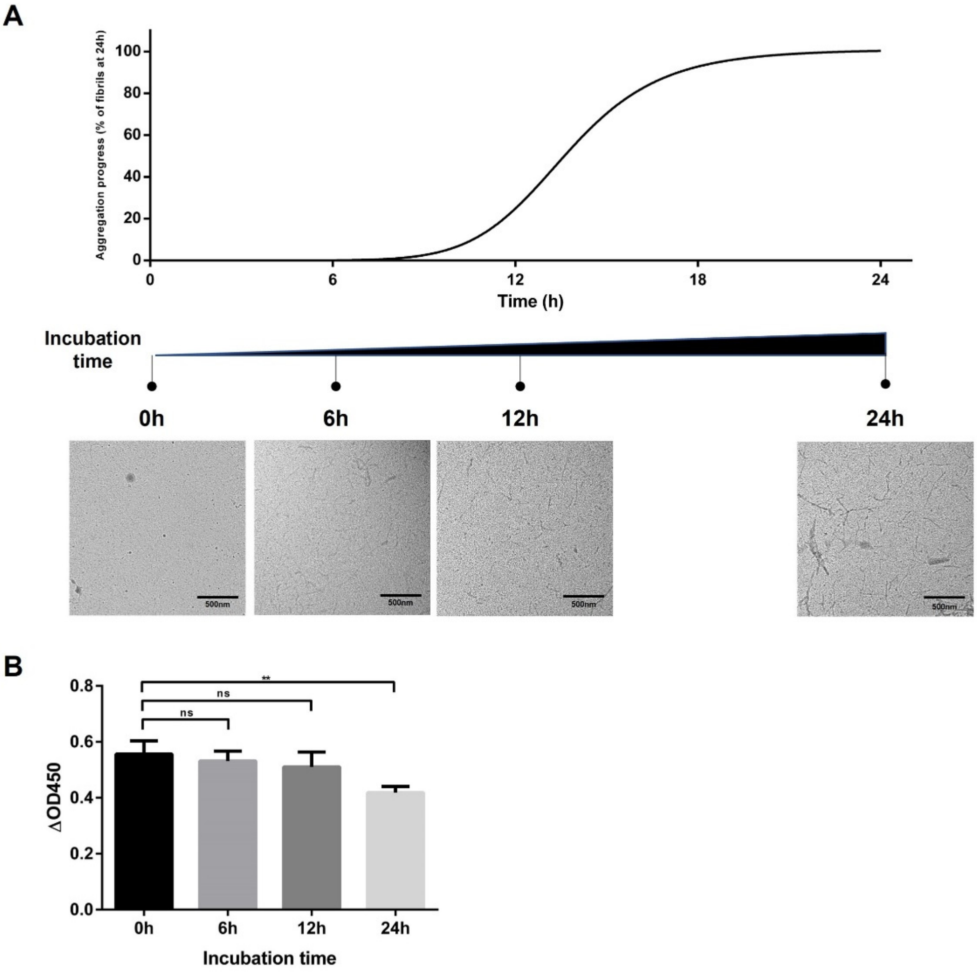

Figure S1. The TEM image of each time point during the amyloid aggregation process and determination of its signal via a developed protocol.

To confirm that the peptide binds to which of the various conformers of Aβ42, we performed TEM and checked the exact state of aggregation of Aβ42 at several time points throughout the aggregation process as described in a previous study [1]. Although the amount of fibril of Ab42 kept increasing with time, the signal by the peptide reached a plateau during the procedure and didn't go up any further, with a slight decline at the end. We think that it is because excessive aggregation of Ab42 makes the peptide hard to bind.

Reference
[1] W. B. Stine, L. Jungbauer, C. Yu, M. J. Ladu, Preparing synthetic Aβ in different aggregation states. Methods Mol Biol. **2011**, 670, 13–32. doi:10.1007/978-1-60761-744-0_2.
